# Exploratory Analysis of Circulating miR‐486‐3p and miR‐21‐5p in Early Detection and Subtype Classification of Luminal A and B Breast Cancer in Peruvian Women

**DOI:** 10.1002/cnr2.70717

**Published:** 2026-10-09

**Authors:** Desireé Bouroncle, Gustavo Colque, Luis Huaytalla‐Bautista, María Claudia Flores‐Berrospi, Carla Bernal, Ivan Belzusarri, Jorge Dunstan, Mónica Paredes‐Anaya

**Affiliations:** ^1^ Genomics and Integrative Biology Laboratory, Faculty of Biological Sciences, Universidad Nacional Mayor de San Marcos Lima Peru; ^2^ Department of Radiotherapy Instituto Nacional de Enfermedades Neoplasicas Lima Peru; ^3^ Department of Breast and Soft Tissue Surgery Instituto Nacional de Enfermedades Neoplasicas Lima Peru

**Keywords:** biomarkers, breast cancer, luminal a, luminal B, microRNAs, miR‐21‐5p, miR‐486‐3p, target genes

## Abstract

**Background:**

Breast cancer (BC) remains the most common malignancy among women worldwide, and early detection is essential for improving clinical outcomes. There is a growing need for accessible, minimally invasive molecular biomarkers that complement current diagnostic approaches and support molecular subtype classification.

**Aims:**

This exploratory case–control study evaluated the plasma expression of four circulating miRNAs (miR‐21‐5p, miR‐486‐3p, miR‐451a, and miR‐660‐5p) in Peruvian women with Luminal A (*n* = 32) and Luminal B (*n* = 43) breast cancer and healthy controls (*n* = 36).

**Methods and Results:**

Expression levels were quantified by qPCR, and diagnostic performance was assessed using logistic regression with leave‐one‐out cross‐validation (LOOCV). miR‐486‐3p was significantly upregulated in Luminal A compared with both controls and Luminal B, whereas miR‐21‐5p was downregulated relative to controls. After validation, miR‐486‐3p retained moderate discriminative ability for distinguishing Luminal A from controls (AUC = 0.651; 95% CI: 0.461–0.841; *p* = 0.120), while the combined miR‐486‐3p/miR‐21‐5p model showed moderate discriminatory performance for differentiating Luminal A from Luminal B (AUC = 0.690; 95% CI: 0.466–0.914; *p* = 0.0959). Exploratory functional enrichment analyses suggested that experimentally validated targets of these miRNAs were associated with cancer‐ and immune‐related pathways, providing biological context for their potential roles in breast cancer.

**Conclusion:**

These findings provide preliminary evidence supporting the potential of circulating miR‐486‐3p and miR‐21‐5p as candidate non‐invasive biomarkers for future early detection and molecular subtype classification of Luminal breast cancer. However, these findings should be interpreted cautiously and require validation in larger, independent cohorts before clinical implementation.

**Abbreviations**
AIC: akaike information criterion
AUC: area under the curve
BC: breast cancer
CON‐CYTEC: National Council of Science, Technology, and Technological Innovation
EMT: epithelial‐mesenchymal transition
ER: estrogen receptor
HER2: human epidermal growth factor receptor 2
INEN: National Institute of Neoplastic Diseases
KEGG: Kyoto Encyclopedia of Genes and Genomes
Ki‐67: marker of proliferation Ki‐67
miRNA: microRNA
oncomiR: oncogenic miRNA
PCONFI‐GI: Research Project Program for Research Groups
PR: progesterone receptor
PRO‐CIENCIA: National Program for Scientific Research and Advanced Studies
ROC: receiver operating characteristic
TNBC: triple‐negative breast cancer

## 1 Introduction

Breast cancer is the most frequently diagnosed malignancy and a leading cause of cancer‐related mortality among women worldwide [1]. In Peru, it remains the most common cancer among women, with a mortality rate of 11.0 per 100 000 inhabitants in 2021 [2], partly due to late detection.

Although mammographic screening plays a central role in early diagnosis, its sensitivity is reduced in younger women and those with dense breast tissue [3], and coverage remains suboptimal in many Latin American countries due to socioeconomic and geographical barriers [4, 5]. These limitations highlight the need for complementary circulating molecular biomarkers that could improve early, non‐invasive diagnosis and molecular classification. Breast cancer comprises multiple molecular subtypes, with Luminal A and Luminal B being the most prevalent hormone receptor–positive forms. Luminal A tumors typically exhibit lower proliferation rates and higher estrogen receptor (ER) expression, while Luminal B tumors present higher Ki‐67 levels, may co‐express HER2, and are associated with poorer prognoses [3]. Given these subtype‐dependent variations in prognosis and treatment response, microRNAs (miRNAs) have emerged as promising non‐invasive biomarkers in oncology [6].

MiRNAs are small, non‐coding RNA molecules that regulate gene expression post‐transcriptionally. Several miRNAs have been implicated in breast cancer biology, including miR‐21‐5p, miR‐486‐3p, miR‐451a, and miR‐660‐5p, each associated with oncogenic or tumor‐suppressive roles [7, 8, 9]. These four candidate miRNAs were selected based on a review of previously published studies identified through PubMed and Google Scholar, covering studies published between 2013 and 2024 and prioritizing circulating miRNAs with reported diagnostic potential in breast cancer, particularly in early‐stage disease and Luminal breast cancer whenever subtype‐specific evidence was available. Circulating miR‐21‐5p has been consistently associated with breast cancer detection and was included in a diagnostic signature for early‐stage disease [10]. Likewise, miR‐486‐3p has been reported to be significantly upregulated in Luminal A breast cancer compared with healthy controls, suggesting subtype‐specific diagnostic potential [11]. miR‐451a has also been proposed as a circulating biomarker and has shown differential expression in breast cancer cohorts [11, 12], whereas miR‐660‐5p has recently emerged as a promising circulating biomarker with increased expression in patients, including patients with early‐stage breast cancer [13].

However, despite these promising findings, important knowledge gaps remain. Previous studies have primarily evaluated circulating miRNAs for breast cancer detection by comparing patients with healthy controls, whereas relatively few have specifically investigated their ability to discriminate between Luminal A and Luminal B subtypes. In addition, evidence is largely derived from non‐Latin American populations, and few studies have focused exclusively on early‐stage disease while incorporating internally validated diagnostic models. Consequently, the clinical value of circulating miRNAs for molecular subtype discrimination remains insufficiently characterized.

To our knowledge, this is one of the first studies to simultaneously evaluate these four circulating miRNAs for both early detection and molecular subtype discrimination between Luminal A and Luminal B breast cancer in a Peruvian cohort. To address these gaps, this exploratory study evaluated the expression profiles and diagnostic performance of four circulating miRNAs (miR‐21‐5p, miR‐486‐3p, miR‐451a, and miR‐660‐5p) in Peruvian women with early‐stage Luminal A and Luminal B breast cancer. Unlike previous studies, our work integrates differential expression analysis while incorporating internal cross‐validation strategies, correlation analysis among candidate circulating miRNAs, and functional pathway enrichment within a single study, providing a more comprehensive characterization of their potential biological and clinical relevance in Luminal breast cancer. By integrating diagnostic modeling, correlation analysis, and pathway enrichment, this study provides preliminary biological insights into the coordinated expression of circulating miRNAs while evaluating their potential clinical utility for early detection and molecular subtype discrimination in Luminal breast cancer.

## 2 Materials and Methods

### 2.1 Study Groups and Biological Material

This observational case–control study focused on early‐stage breast cancer and included 36 control subjects without a family history of breast cancer, 32 patients with early‐stage (I–II) Luminal A tumors, and 43 patients with early‐stage (I–II) Luminal B tumors, all recruited before treatment. All participants were recruited from the National Institute of Neoplastic Diseases (INEN) in Lima, Peru. The diagnosis of all cases was confirmed through histological and immunohistochemical analyses. According to the criteria used at INEN, ER positivity was defined as ≥ 1%, PR positivity as ≥ 20%, Ki‐67 expression was categorized as low (< 15%) or high (≥ 15%), and HER2 status was classified as positive or negative (Table S1). Luminal A tumors were defined as ER‐ and PR‐positive, HER2‐negative tumors with low Ki‐67 expression (< 15%). Luminal B tumors were defined as ER‐positive tumors with any PR status, HER2‐positive or HER2‐negative status, and high Ki‐67 expression (≥ 15%). Following the Helsinki Declaration, this study received ethical approval from the INEN Ethical Committee and the Faculty of Biological Sciences at the National University of San Marcos. All participants were informed about the study's objectives and procedures and provided written informed consent. Peripheral blood samples were collected from each participant, along with relevant clinical and demographic data, using a standardized data collection form.

Luminal B breast tumors can be further classified into HER2‐positive and HER2‐negative subtypes. In the present study, Luminal B cases included both HER2‐positive and HER2‐negative tumors, which were analyzed as a single group.

### 2.2 Plasma Isolation

Peripheral blood (3 mL) was collected into K2‐EDTA Vacutainer tubes at INEN and transported under a maintained cold chain (4°C) to the laboratory. Plasma separation was performed within 3 h of blood collection. Each sample was divided into three 1.5 mL Eppendorf tubes and centrifuged at 1800*g* for 10 min at 4°C using a refrigerated centrifuge. The supernatant plasma was transferred to new 1.5 mL tubes and centrifuged again at 20000*g* for 10 min at 4°C to remove residual cellular debris. The resulting plasma was carefully collected, avoiding the pellet, aliquoted in 270 μL volumes, and stored at −80°C until further analysis. Each aliquot was thawed only once before RNA extraction.

### 2.3 Total RNA Extraction

Total RNA, including miRNAs, was extracted from 200 μL of plasma samples using the Plasma/Serum RNA Purification Mini Kit (Cat. No. 55000, Norgen Biotek Corp., Thorold, Ontario, Canada), following the manufacturer's instructions. Prior to extraction, plasma aliquots were thawed on ice and allowed to equilibrate for approximately 1–1.5 h at 4°C to ensure complete thawing while minimizing RNA degradation. All procedures were performed under cold conditions (~4°C) to preserve RNA integrity. RNA concentration was quantified using a NanoDrop spectrophotometer (Thermo Fisher Scientific, Waltham, MA, USA), and samples were stored at −80°C until reverse transcription.

### 2.4 Primer Design

Primers were designed using the miRprimer software tool [14], and their sequences are presented in Table S2.

### 2.5 Polyadenylation and Reverse Transcription

Polyadenylation and reverse transcription of the extracted RNA were performed following the protocol described by Cirera and Busk [15]. The reverse transcription master mix was prepared using the following components: 2 μL of 10× *E. coli* poly(A) polymerase reaction buffer (New England Biolabs, USA), 2 μL of ATP (1 mM) (New England Biolabs, USA), 2 μL of RT primer (10 μM), 2 μL of dNTP mix (1 mM) (Promega, USA), 0.5 μL of MultiScribe Reverse Transcriptase (50 U/μL), 0.2 μL of *E. coli* poly(A) polymerase (5000 U/mL) (New England Biolabs, USA), and 1.3 μL of RNase‐free water. Finally, 10 μL of RNA at a standardized concentration of 2 ng/μL (20 ng total) was added to the reaction. Samples with higher RNA concentrations were diluted to this working concentration prior to reverse transcription. The reaction was incubated at 42°C for 1 h, followed by enzyme inactivation at 95°C for 5 min using a Mastercycler Nexus Thermal Cycler (Eppendorf, Hamburg, Germany). The synthesized cDNA was stored at −20°C until further use.

### 2.6 Expression Analysis

MiRNA expression levels were assessed using miRNA‐specific quantitative PCR (qPCR) with the qPCRBIO SyGreen Mix Hi‐ROX kit (PCR Biosystems, London, UK), and qPCR reactions were carried out on a StepOnePlus Real‐Time PCR System (Applied Biosystems, Foster City, CA, USA). Amplification reactions were prepared in a final volume of 10 μL, consisting of 5 μL of master mix, 0.4 μL of each primer, 0.1 μL of ROX, 1.1 μL of molecular‐grade water, and 3 μL of cDNA diluted 1:6. Cycling conditions followed the manufacturer's protocol: 95°C for 2 min, followed by 40 cycles of 95°C for 5 s and 60°C for 30 s. Each plasma sample was analyzed in technical duplicates, and no‐template controls (NTC) were included for each miRNA in every 96‐well plate to monitor potential contamination or non‐specific amplification. Primer efficiency and linearity were evaluated through standard curves, with efficiencies ranging from 86% to 113% and slopes between −3 and −3,7 for most assays; miR‐660‐5p displayed an efficiency outside the optimal range and was interpreted with caution. Samples with Ct values greater than 36 were excluded from further analysis. To minimize inter‐plate variability, an inter‐plate calibrator consisting of pooled plasma samples was included on all plates, and correction factors derived from this calibrator were applied to harmonize runs. Amplification specificity was assessed by melt‐curve analysis following each RT‐qPCR run (see Figure S1). RNU6 (U6 small nuclear RNA) was used as the endogenous reference gene for ΔCt normalization, following previous reports in plasma miRNA studies [16]. In the present cohort, RNU6 exhibited consistent Ct values across the study groups, with no significant differences between controls, Luminal A, and Luminal B samples, supporting its use as the endogenous reference in the present study. Fold‐change was calculated using the 2^−ΔΔCt^ method [17].

### 2.7 Statistical Analysis

All statistical analyses were performed using R version 4.4.1 [18]. Data wrangling and preprocessing were carried out using the packages dplyr (v1.1.4) and tidyr (v1.3.0). For group comparisons, normality was assessed with the Shapiro–Wilk test (shapiro.test, base R). Categorical variables were compared among groups using the Chi‐square test. Continuous variables were analyzed using one‐way ANOVA for normally distributed data or Kruskal–Wallis for non‐normal data. Post hoc analyses were performed using multcomp (v1.4–25) for Tukey's test and dunn.test (v1.3.5) for Dunn's test, with Bonferroni correction applied in both cases. Boxplots with annotated *p*‐values were generated using ggplot2 (v3.5.1) and ggpubr (v0.6.0).

Correlations between numerical variables were evaluated using either Pearson or Spearman correlation coefficients, depending on the distribution. Spearman's *ρ* and associated *p*‐values were computed using Hmisc (v5.1‐3) (rcorr). Correlation heatmaps were produced with ggcorrplot (v0.1.4), and scatterplot matrices with trend lines using GGally (v2.2.1). Visualizations were generated with ggplot2, and color mapping with scales (v1.3.0).

Receiver Operating Characteristic (ROC) curve analyses were performed using pROC (v1.18.5) [19], with AUC values and 95% confidence intervals computed by DeLong's method. Leave‐one‐out cross‐validation (LOOCV) was implemented for model validation, using ROCR (v1.0‐11) [20] for AUC estimation on out‐of‐fold predictions. Logistic regression models (glm, family = binomial) were fit with base R. For multi‐marker models, the expression values of selected miRNAs were entered as predictors in a multivariable logistic regression model, and the predicted probabilities were used as continuous scores for ROC curve construction. For single‐marker models, ROC curves were generated directly from the expression values.

To minimize overfitting, leave‐one‐out cross‐validation (LOOCV) was applied. LOOCV was selected because this exploratory study involved a relatively limited sample size, allowing each model to be trained using all available observations except one in each iteration while providing an internal estimate of model performance. This approach maximizes the use of the available data and reduces information loss compared with conventional train‐test splits, making it suitable for exploratory biomarker studies without an independent validation cohort. To address zero‐cell issues, a continuity correction of 0.5 was applied to TP, FP, TN, and FN counts when deriving diagnostic metrics (sensitivity, specificity, PPV, NPV, and accuracy). A two‐tailed *p* < 0.05 was considered statistically significant. Continuous data were presented as mean ± standard deviation (SD) for normally distributed variables, or as median [interquartile range, IQR] otherwise. Categorical variables were summarized as counts and percentages.

### 2.8 miRNAs Target Prediction and Functional Analysis

The experimentally validated gene targets for the miRNAs of interest (hsa‐miR‐21‐5p and hsa‐miR‐486‐5p) were obtained using the multiMiR package [21] in the R statistical environment (version 4.4.1). The get_multimir() function was used, specifying the parameter table = “validated” to retrieve only interactions supported by experimental evidence. The results were subsequently filtered to retain only those interactions with positive or functional support (support_type = “positive” or “Functional MTI”), according to the classification provided by the multiMiR database. This analysis yielded a refined list of genes with direct experimental validation using reporter assays, microarrays, qPCR, and other associated functional techniques. The sets of targets obtained for hsa‐miR‐21‐5p and hsa‐miR‐486‐5p were subsequently compared to determine the existence of shared targets.

The complete list of experimentally validated target genes was subsequently used for exploratory functional and pathway enrichment analyses (see Supporting Information). These analyses were intended to generate biological hypotheses regarding potential pathways associated with the candidate miRNAs and were not designed to provide mechanistic validation.

Pathway enrichment analysis was performed in R (version 4.4.1) using the clusterProfiler package [22], available in Bioconductor. This package allows gene sets to be compared with up‐to‐date biological databases, such as the Kyoto Encyclopedia of Genes and Genomes (KEGG). For each miRNA (hsa‐miR‐21‐5p and hsa‐miR‐486‐5p), the experimentally validated target gene sets obtained using the multiMiR package were used. Gene names were converted to Entrez codes, which were used as input to the enrichKEGG() function, specifying the organism *Homo sapiens* (“hsa”) and setting a significance threshold of *p*‐value < 0.05.

The analysis generated a table with the significantly enriched pathways, including their KEGG ID, description, gene ratio (GeneRatio), adjusted *p*‐value (p.adjust), and associated genes. The results were then visualized using the dotplot() function of the clusterProfiler package, which graphically represents the top enriched pathways. The bubble size reflects the number of associated genes, and the color indicates the adjusted *p*‐value. Finally, the 20 most significant pathways for hsa‐miR‐21‐5p and the two significant pathways for hsa‐miR‐486‐5p were selected. In both cases, the enriched pathways were interpreted according to their biological relevance and their potential association with cancer‐related and immune‐related signaling pathways. These findings were considered hypothesis‐generating and intended to provide biological context rather than evidence of direct mechanistic relationships.

## 3 Results

### 3.1 Anthropometric Characteristics and Data of the Study Groups

A total of 111 women were included and classified into three groups: controls (*n* = 36), Luminal A (*n* = 32), and Luminal B (*n* = 43). Anthropometric and gynecologic data were obtained from clinical records. The general characteristics of the participants are summarized in Tables 1 and S3. The distribution of anthropometric and gynecologic variables across the study groups is shown in Figure S2. No significant differences were observed among groups for most evaluated variables (*p* > 0.05), except for family history of breast cancer, which was statistically significant in Luminal A and B groups compared with controls (*p* = 1.042e−07, Table S3).

**TABLE 1:** Comparison of anthropometric and gynecologic variables among Peruvian women with Luminal A and B breast cancer and healthy controls.

Variable	Control (*n* = 36)	Luminal A (*n* = 32)	Luminal B (*n* = 43)	*p*‐value	Test used
Age (years)	51.8 ± 10.29	54.31 ± 11.25	52.07 ± 10.29	0.584	ANOVA
Weight (kg)	61.50 (47–90)	68 (40–81)	62.50 (43–87)	0.44	Kruskal‐Wallis
Height (m)	1.55 ± 0.05	1.54 ± 0.06	1.54 ± 0.06	0.549	ANOVA
BMI (kg/m^2^)	24.85 (18–36.98)	27.96 (18.82–34.67)	26.67 (19.11–38.67)	0.213	Kruskal‐Wallis
Menarche age (years)	12.00 (9.00–18.00)	13.00 (9.00–16.00)	12.00 (9.00–16.00)	0.729	Kruskal‐Wallis
Age at first pregnancy (years)	25.50 (0.00–38.00)	22.50 (0.00–40.00)	22.00 (17.00–43.00)	0.830	Kruskal‐Wallis
Number of pregnancies	2.00 (0.00–7.00)	3.00 (0.00–9.00)	2.00 (0.00–8.00)	0.121	Kruskal‐Wallis

*Note:* Normally distributed variables are expressed as Mean ± SD; non‐normally distributed variables are expressed as Median (Min–Max). Statistical significance was set at *p* < 0.05.

Abbreviations: BMI, body mass index; *n*, sample size; SD, standard deviation.

### 3.2 Differential Expression of miR‐486‐3p and miR‐21‐5p Among Study Groups

Most circulating miRNAs did not follow a normal distribution across study groups (*p* < 0.05), except for miR‐486‐3p, which shows approximate normality in the Control and Luminal B groups. Therefore, non‐parametric tests were applied for all comparisons. Among the four miRNAs analyzed, miR‐21‐5p was significantly downregulated in Luminal A patients compared with Controls (7.4‐fold decrease; *p* = 0.0104, Figure 1A). In contrast, miR‐451a showed no significant differences among groups (Figure 1B). Since miR‐451a is commonly used as a marker of hemolysis, the absence of inter‐group variation suggests that sample hemolysis was minimal and likely did not substantially affect the quantification of the remaining circulating miRNAs.

**FIGURE 1:**
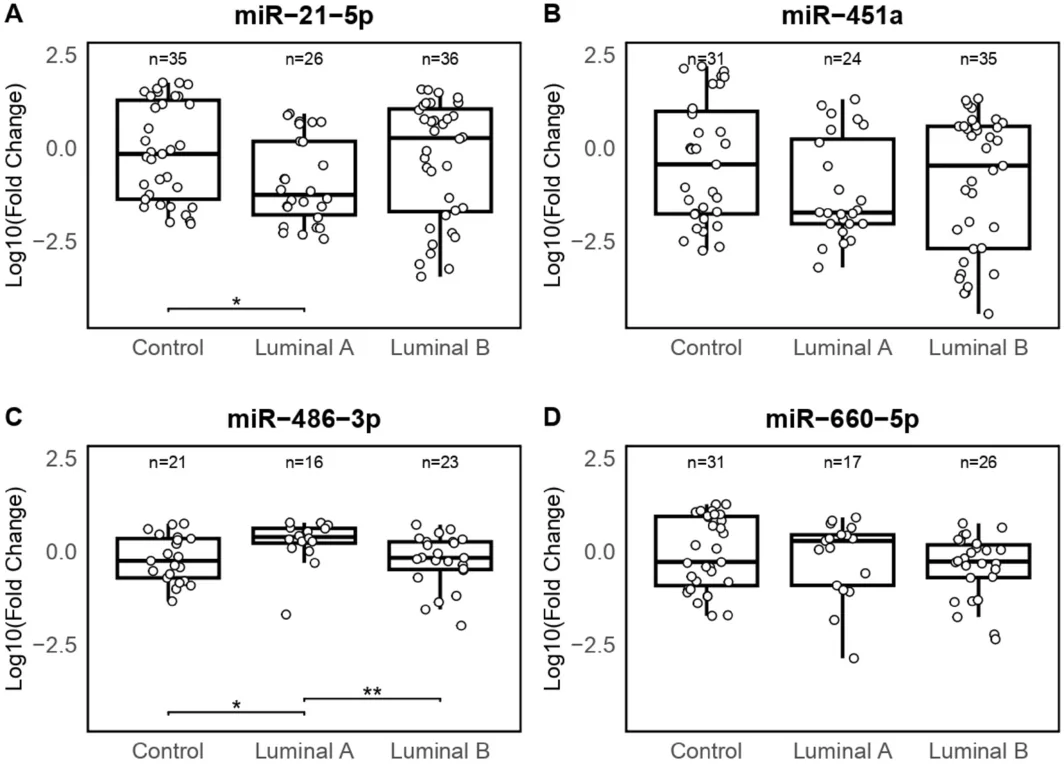
Comparison of miRNA expression levels among breast cancer subtypes and the control group for miR‐21‐5p (A), miR‐451a (B), miR‐486‐3p (C), and miR‐660‐5p (D). Expression levels were calculated as Log_10_ (Fold Change) relative to the control group (reference sample). Pairwise comparisons were performed using Dunn's test with Bonferroni correction, and the reported *p*‐values correspond to Bonferroni‐adjusted *p*‐values. *p ≤ 0.05, **p ≤ 0.01.

miR‐486‐3p was upregulated in Luminal A compared with both Controls (*p* = 0.0166) and Luminal B patients (*p* = 0.00195, Figure 1C). Finally, miR‐660‐5p shows no significant expression differences across groups (Figure 1D). Taken together, these findings indicate that miR‐486‐3p and miR‐21‐5p exhibited the most distinct expression profiles between Luminal A and Controls; meanwhile, no statistically significant differences were observed among Luminal A, Luminal B, and control groups for miR‐451a and miR‐660‐5p.

### 3.3 Correlation Analysis of miRNA Expression

Spearman correlation analysis was performed to assess the relationships among miRNA expression levels. A strong positive correlation (*ρ* = 0.82, *p* < 0.05) was observed between miR‐451a and miR‐21‐5p across the Luminal A and control groups (Figure 2), suggesting potential co‐regulation.

**FIGURE 2:**
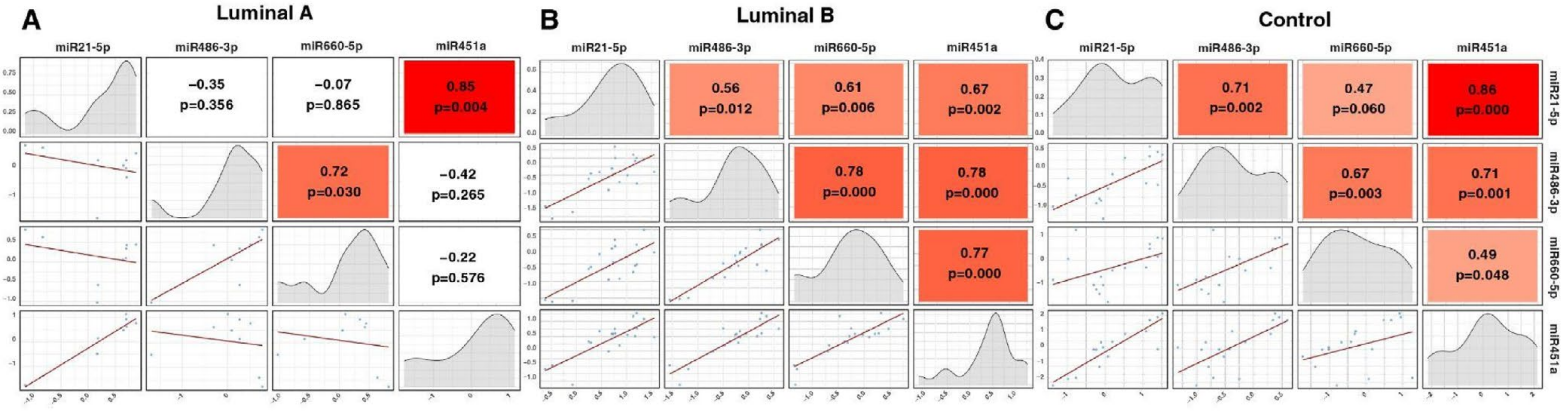
Spearman correlation matrices showing pairwise relationships between the four circulating miRNAs within each group: (A) Luminal A, (B) Luminal B, and (C) control group. The color scale represents the strength and direction of the correlation coefficient (*ρ*), with red indicating a positive correlation and color intensity proportional to the absolute value of *ρ*. Reported *p*‐values correspond to the statistical significance of each correlation (*p* < 0.05).

Additionally, correlations were evaluated between the four circulating miRNAs and a set of clinico‐pathological and lifestyle variables within each group (see Figure S3). No significant associations were detected, except for miR‐21‐5p, which shows a positive correlation with body weight in the control group (Figure S3C, *ρ* = 0.53, *p* < 0.01).

### 3.4 ROC Curve and AUC Evaluation

ROC curve analyses were performed exclusively for miR‐21‐5p and miR‐486‐3p, as these were the only miRNAs that showed statistically significant expression differences between groups (Figure 3A–C; Table S4). In the Luminal A vs. Control comparison, miR‐486‐3p demonstrated the highest performance (AUC = 0.651; 95% CI: 0.461–0.841; *p* = 0.120) after LOOCV validation, maintaining a comparable AUC value to that obtained before validation (AUC = 0.717; Table S5), although its *p*‐value lost statistical significance (Figure 3A; Tables S5 and S6). For the Luminal B vs. Control comparison, the combination of both miRNAs achieved the highest AUC (AUC = 0.635; 95% CI: 0.460–0.810; *p* = 0.132) after LOOCV validation, compared with an apparent AUC of 0.600 (Table S5). The *p*‐value remained non‐significant (Figure 3B; Tables S5–S5). In the Luminal A vs. Luminal B comparison, the combined model of both miRNAs reached an AUC of 0.690 (95% CI: 0.466–0.914; *p* = 0.0959) after LOOCV, compared to the value before validation (AUC = 0.790; Table S5). Before validation, the model showed a statistically significant *p*‐value (*p* = 0.007), in contrast with the non‐significant *p*‐value obtained after LOOCV (Figure 3C; Table S5).

**FIGURE 3:**
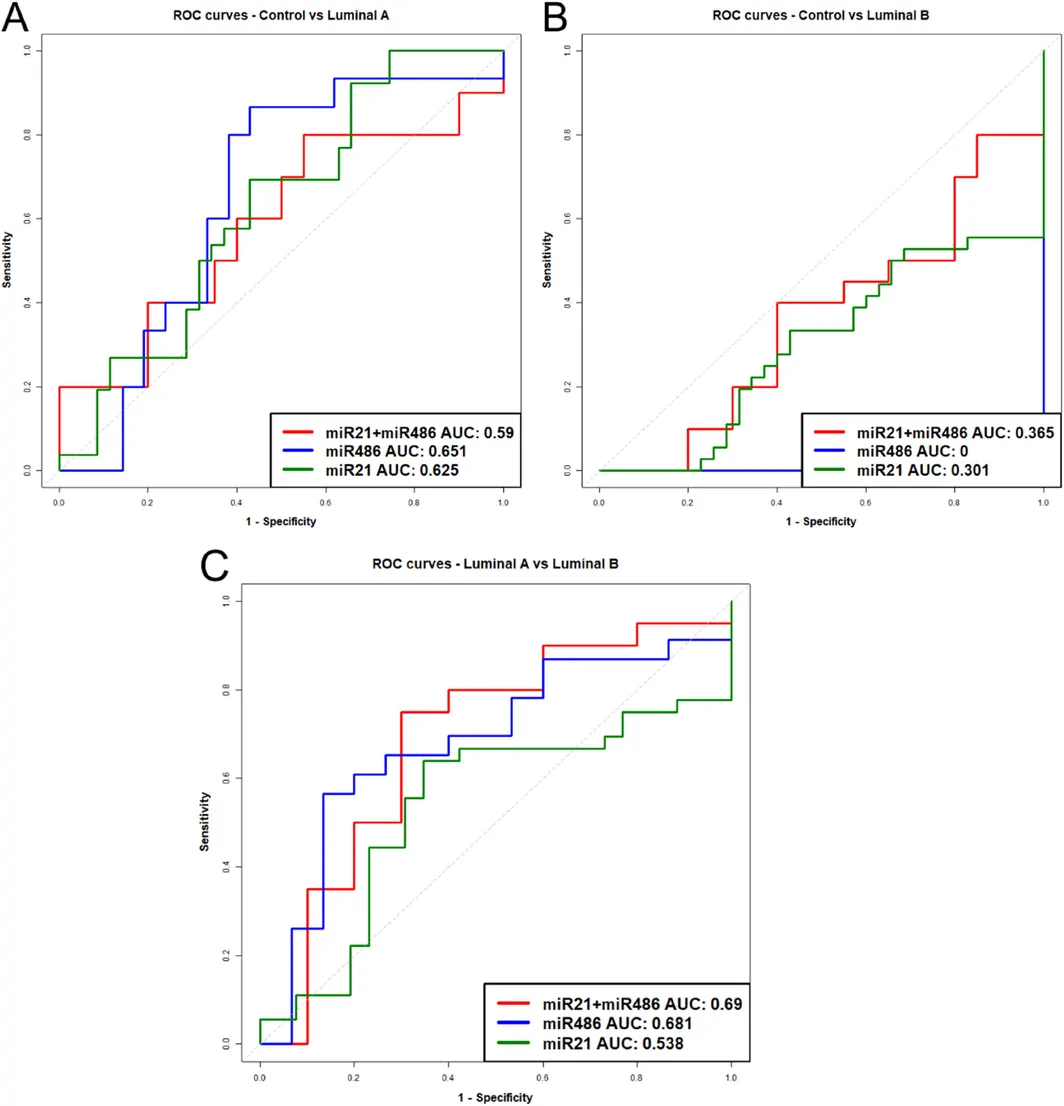
ROC curves generated from LOOCV‐validated logistic regression models based on the expression levels of miR‐21‐5p and miR‐486‐3p. The AUC values shown correspond to LOOCV‐validated model performance. (A) Control vs. Luminal A, (B) Control vs. Luminal B, and (C) Luminal A vs. Luminal B.

Overall, these AUC values indicate a moderate discriminatory capacity, suggesting that miR‐486‐3p and miR‐21‐5p retain biologically relevant classification potential, even after cross‐validation.

Given the potential of miR‐486‐3p and miR‐21‐5p to discriminate Luminal A from Luminal B tumors, we integrated these miRNAs with conventional clinical markers to assess their joint discriminatory power. The clinical markers ER, PR, HER2, and Ki‐67 were included for comparative purposes, serving as benchmarks to evaluate the performance of the miRNA‐based models against established immunohistochemical predictors.

As shown in Figure 4, the addition of Ki‐67 to the miR‐486‐3p and miR‐21‐5p model increased the AUC from 0.690 (95% CI: 0.466–0.914; *p* = 0.0959) to 0.785 (95% CI: 0.551–1.000; *p* = 0.0128) after LOOCV validation (Table S6). However, this combined model did not outperform Ki‐67 alone, which achieved a higher AUC of 0.855. The three‐marker model showed an AUC comparable to that of the classical clinical panel consisting of ER, PR, HER2, and Ki‐67 (AUC = 0.750). In contrast, adding ER, PR, and HER2 to the miRNA‐based models resulted in lower AUC values (0.660–0.670) (Figures 4 and S5; Tables S6 and S7).

**FIGURE 4:**
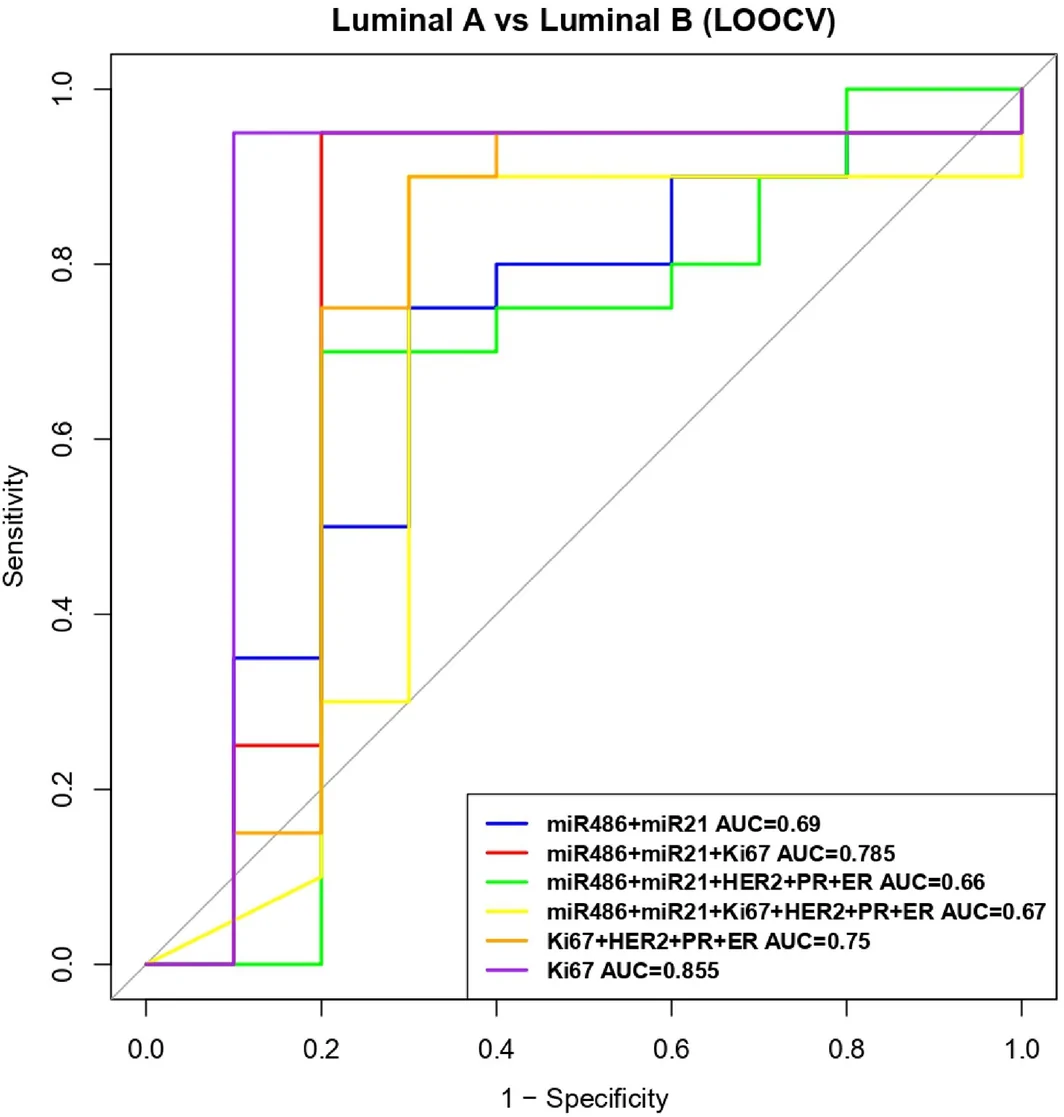
ROC curves of LOOCV‐validated classification models for distinguishing Luminal A from Luminal B tumors. The models included miR‐486‐3p and miR‐21‐5p, alone or in combination with other clinicopathological markers. The reported AUC values correspond to LOOCV‐validated model performance.

### 3.5 Functional Enrichment of Predicted miRNA Targets

A total of 135 experimentally validated gene targets for hsa‐miR‐21‐5p and six validated targets for hsa‐miR‐486‐5p were identified. These interactions correspond to genes whose regulation has been confirmed through functional assays and positive experimental evidence, according to the information integrated in the multiMiR databases. In this analysis, only targets from the experimentally validated category were considered. We also explored whether both miRNAs shared any experimentally validated targets; however, no common genes were identified (Tables S8 and S9).

Exploratory KEGG pathway enrichment analysis was performed using the sets of experimentally validated target genes for each miRNA. For hsa‐miR‐21‐5p, a total of 20 significantly enriched pathways were identified, most of which were associated with cancer and immune‐related processes. The top enriched pathways included MiRNAs in cancer, MAPK signaling pathway, TNF signaling pathway, Proteoglycans in cancer, AGE‐RAGE signaling in diabetic complications, and Platinum drug resistance (Figure 5A). These findings suggest that experimentally validated targets of miR‐21‐5p are enriched in pathways associated with tumorigenic and inflammatory signaling, providing biological context for its potential role in breast cancer.

**FIGURE 5:**
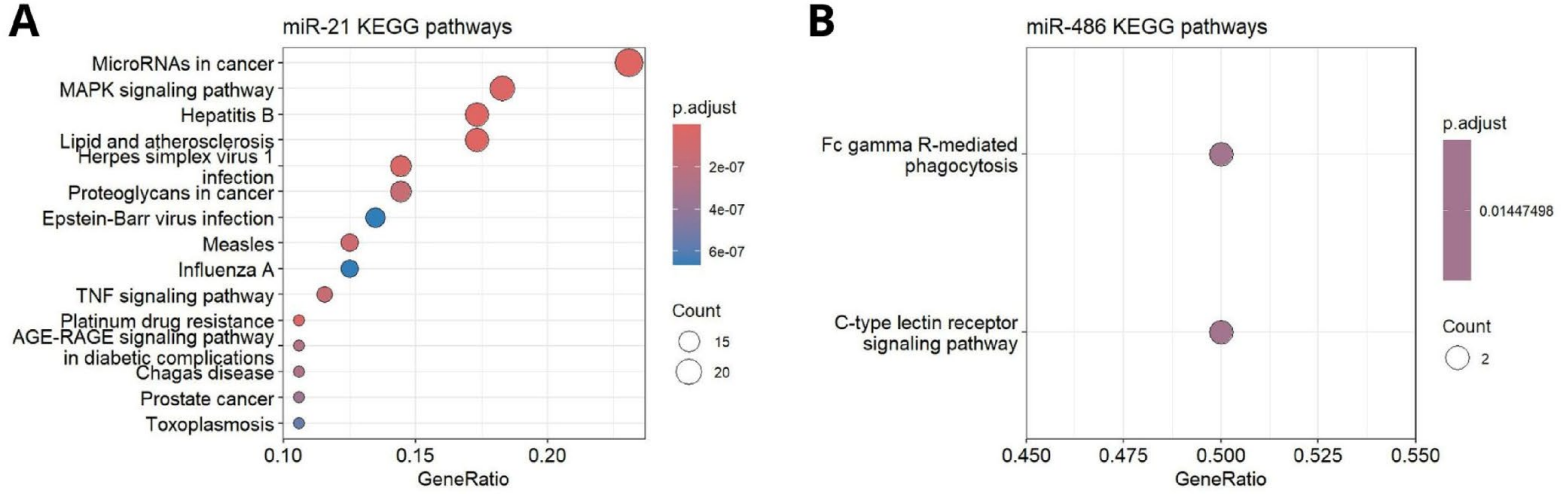
KEGG pathway enrichment analysis for hsa‐miR‐21‐5p and hsa‐miR‐486‐5p. Bubble plots showing significantly enriched KEGG pathways for experimentally validated target genes of each miRNA: (A) hsa‐miR‐21‐5p and (B) hsa‐miR‐486‐5p. The size of the bubbles represents the number of genes involved (Count) and the color indicates the adjusted *p* value (p.adjust).

In contrast, hsa‐miR‐486‐5p was enriched in only two pathways: Fc gamma R‐mediated phagocytosis and C‐type lectin receptor signaling pathway (Figure 5B), both related to immune modulation and phagocytic activity. Importantly, no KEGG pathways were shared between miR‐21‐5p and miR‐486‐5p, suggesting that these miRNAs may be associated with distinct biological processes and signaling pathways.

## 4 Discussion

This study evaluated four circulating miRNAs (miR‐21‐5p, miR‐486‐3p, miR‐451a, and miR‐660‐5p) as potential circulating, non‐invasive biomarkers for early‐stage Luminal breast cancer in a Peruvian cohort. Among them, miR‐486‐3p showed the greatest potential for distinguishing Luminal A from both Luminal B tumors and healthy controls, whereas miR‐21‐5p primarily contributed to differentiating Luminal A patients from controls (Figure 1). Functional enrichment analyses suggested that experimentally validated targets of both miRNAs were enriched in cancer‐related pathways, providing biological context for their potential roles in breast cancer. As these analyses were based on previously validated target genes, they should be interpreted as hypothesis‐generating rather than evidence of direct mechanistic relationships.

Our results support a role for miR‐486‐3p as a potential classifier distinguishing Luminal A breast cancer from both healthy controls and Luminal B subtypes. Similar findings regarding its upregulation in Luminal A compared with controls were reported by Arabkari et al. [11], reinforcing its involvement in Luminal A tumor biology. However, previous studies have reported conflicting roles for miR‐486‐3p, which functions either as an oncomiR or a tumor suppressor depending on the cancer type and context [11, 23]. Such divergent effects are common among miRNAs and often reflect context‐dependent regulation. In our study, miR‐486‐3p showed a statistically significant ability to discriminate Luminal A from controls in the apparent model (AUC = 0.717, *p* = 0.0166); however, this significance was lost after LOOCV validation (AUC = 0.651, *p* = 0.120). Similarly, the comparison between Luminal A and Luminal B shows only a marginal trend toward significance (*p* = 0.0505). These results suggest that the initial discriminatory performance of miR‐486‐3p may have been influenced by overfitting or sample‐specific variation. Nonetheless, its consistent upregulation pattern across comparisons supports its potential as a candidate biomarker that requires validation in larger independent cohorts.

Contrary to most reports describing miR‐21‐5p upregulation in breast cancer [10], our cohort shows reduced expression in Luminal A patients compared with controls. Previous studies have linked miR‐21‐5p overexpression to the inhibition of apoptosis through the suppression of PTEN and PDCD4 [24]. As all patients in our study presented early‐stage (I–II) disease, this downregulation may reflect molecular features characteristic of early Luminal A tumors. Additionally, differences in hormone receptor status may contribute to this pattern, as estradiol has been shown to suppress miR‐21‐5p expression in ER‐positive tumors [25, 26]. These findings underscore the need for regional validation of circulating biomarkers, since molecular signatures can vary across patient populations and disease contexts.

Overall, the combination of miR‐21‐5p and miR‐486‐3p achieved moderate discriminatory performance for Luminal A cases, comparable to previously reported miRNA panels [11]. Although the sample size was modest, internal validation using LOOCV resulted in lower AUC values and loss of statistical significance for several models, providing a more conservative estimate of their predictive performance (Figures 3 and S4). This pattern is consistent with the purpose of internal validation, which aims to reduce optimism bias and provide a more realistic assessment of model generalizability in exploratory studies with limited sample sizes. Nevertheless, miR‐486‐3p retained moderate discriminative performance after validation, supporting its potential as a candidate biomarker that warrants further evaluation in larger, independent cohorts. The addition of Ki‐67 to the miR‐486‐3p and miR‐21‐5p model increased the AUC from 0.690 to 0.785. However, this combined model did not outperform Ki‐67 alone, which achieved a higher AUC of 0.855 (Figures 4 and S5; Tables S6 and S7). The three‐marker model showed performance comparable to that of the classical clinical panel (ER, PR, HER2, and Ki‐67; AUC = 0.750). This finding is noteworthy given the known limitations of Ki‐67 scoring, including inter‐laboratory variability and the lack of standardized cutoffs [27]. Although the circulating miRNAs did not improve discrimination beyond Ki‐67 alone in the present study, their potential complementary role in future subtype classification strategies warrants further investigation. However, these findings require validation in larger, independent cohorts.

Neither miR‐451a nor miR‐660‐5p shows significant expression differences across groups (Figure 1), although both have been reported as dysregulated in various cancers [13, 28, 29, 30]. Variability in results across studies may stem from technical differences in RNA extraction, quantification thresholds, or sample heterogeneity. The stable expression of miR‐451a in our samples may also indicate adequate hemolysis control, as this miRNA is highly abundant in erythrocytes and its elevation is typically associated with red‐blood‐cell contamination [31]. Interestingly, we observed a strong positive correlation between miR‐451a and miR‐21‐5p across all groups (Figure 2), suggesting potential co‐regulation, possibly through shared involvement in the Wnt/β‐catenin signaling pathway [32, 33]. This association has been documented in other tumor models, warranting further research into their combined roles in breast cancer pathogenesis.

Exploratory analyses of experimentally validated target genes identified distinct biological contexts for miR‐21‐5p and miR‐486‐3p. KEGG enrichment associated miR‐21‐5p targets with tumorigenesis‐ and inflammation‐related pathways, including MiRNAs in cancer, MAPK signaling, and TNF signaling [34, 35], whereas miR‐486‐3p targets were enriched in immune‐related pathways involved in Fc gamma receptor‐mediated phagocytosis and C‐type lectin receptor signaling [36, 37]. These findings are consistent with previous evidence linking miR‐21‐5p to PTEN suppression and PI3K/AKT/mTOR signaling [38, 39, 40], while miR‐486‐3p has been indirectly associated with PTEN regulation in other malignancies [41, 42]. Together, these exploratory analyses provide biological context for the observed expression patterns and suggest that both miRNAs may participate in complementary proliferative and immune‐related processes in Luminal breast cancer. However, these observations should be considered hypothesis‐generating because they were not functionally validated in the present study.

The principal limitation of this study is the sample size, which may have reduced statistical power and the ability to detect subtle differences for miR‐451a and miR‐660‐5p. Another important limitation is the absence of a prospective hemolysis control; however, miR‐451a, commonly used as a hemolysis control miRNA, did not differ significantly between groups, suggesting that the impact of hemolysis on miRNA comparisons was minimal. The absence of no‐RT controls is also a methodological limitation, as these controls could help assess potential genomic DNA contamination. This should still be addressed in future studies to meet current best practices in circulating miRNA analysis. Future studies could also evaluate multiple endogenous reference genes and/or exogenous spike‐in controls to further strengthen normalization strategies for circulating miRNA analyses.

Overall, circulating miR‐486‐3p and miR‐21‐5p showed promising, although preliminary, potential as candidate biomarkers for early‐stage Luminal breast cancer. While miR‐21‐5p primarily contributed to distinguishing Luminal A tumors from healthy controls, miR‐486‐3p also showed potential for molecular subtype discrimination. Integration of these circulating biomarkers with conventional clinicopathological markers may support future minimally invasive strategies for early detection and subtype classification. However, validation in larger independent cohorts is required before clinical implementation.

## Author Contributions

**Desireé Bouroncle:** methodology, software, validation, formal analysis, investigation, data curation, writing – original draft, visualization, funding acquisition. **Gustavo Colque:** methodology, software, validation, formal analysis, investigation, data curation, writing – original draft. **Luis Huaytalla‐Bautista:** methodology, software, validation, formal analysis, investigation, writing – original draft. **María Claudia Flores‐Berrospi:** software, investigation, writing – original draft. **Carla Bernal:** conceptualization, methodology, writing – review and editing. **Ivan Belzusarri:** resources, writing – review and editing, supervision. **Jorge Dunstan:** resources, writing – review and editing, supervision. **Mónica Paredes‐Anaya:** conceptualization, resources, writing – review and editing, supervision, project administration, funding acquisition.

## Funding

This research was funded by the National Council of Science, Technology, and Technological Innovation (CONCYTEC) and the National Program for Scientific Research and Advanced Studies (PROCIENCIA) within the framework of Competition E073‐2024‐01 for Undergraduate and Graduate Theses in Science, Technology, and Technological Innovation (Grant No PE501089659‐2024). It also received support from CONCYTEC through the PROCIENCIA program, under the call “Internships in science, technology, and technological innovation—E009‐2024‐01”, according to contract No. PE501091156‐2024. This study also received funding from the Universidad Nacional Mayor de San Marcos through financial support from the Research Project Program for Research Groups (GI) PCONFIGI 2024 [Project Code: B24101631].

## Ethics Statement

The study was conducted in accordance with the Declaration of Helsinki, and approved by the Ethics Committee of the Faculty of Biological Sciences, National University of San Marcos (protocol code No. 071‐2024‐CBE‐FCB‐UNMSM, date of approval: April 25, 2024) and by the Ethics Committee of Instituto Nacional de Enfermedades Neoplásicas (INEN 18‐90, 11/27/2018). Informed consent was obtained from all subjects involved in this study. Written informed consent has been obtained from the patients to publish this paper.

## Conflicts of Interest

The authors declare no conflicts of interest. The funders had no role in the design of the study; in the collection, analyses, or interpretation of data; in the writing of the manuscript; or in the decision to publish the results.

## Supporting information


**Figure S1:** Representative melt‐curve analysis of the RT‐qPCR assays. Melt curves were generated for (A) miR‐21‐5p, (B) miR‐451a, (C) miR‐486‐3p, (D) miR‐660‐5p, and (E) RNU6. Each curve represents individual samples included in a representative qPCR plate. A predominant melting peak was observed for each target. Melt‐curve analysis was performed for all qPCR runs.
**Figure S2:** Comparison of non‐genetic factors between breast cancer subtypes and the control group. (A) Boxplot comparing BMI across the Control, Luminal A, and Luminal B groups. (B) Boxplot showing the distribution of age among groups. (C) Boxplot comparing the age at menarche. (D) Boxplot illustrating differences in age at first pregnancy. (E) Boxplot comparing the number of pregnancies between groups. (F) Bar plot showing the proportion of individuals with a family history of cancer across groups. (G) Bar chart showing the proportion of postmenopausal women among different groups.
**Figure S3:** Spearman correlation heatmaps between circulating miRNA expression levels and clinico‐pathological/lifestyle variables across breast cancer subtypes and controls. (A) Luminal A group; (B) Luminal B group; (C) Control group; (D) Combined Luminal A + B groups. The color scale represents the strength and direction of the correlation (red = positive, blue = negative). Correlations were computed using Spearman's rank method. Binary variables such as Smoker and Family History of Cancer are shown for completeness, but their interpretation is limited by small sample sizes (e.g., only three smokers).
**Figure S4:** Evaluation of AUC values for predictive models based on miR‐21‐5p and miR‐486‐3p (apparent performance, before validation). (A) Control vs. Luminal A, (B) Control vs. Luminal B, and (C) Luminal A vs. Luminal B.
**Figure S5:** ROC curve analysis comparing the predictive performance of miR‐486‐3p and miR‐21‐5p combined with HER2, PR, ER, and Ki‐67 in distinguishing Luminal A from Luminal B breast cancer subtypes (*p* < 0.05).

**Table S1:** Clinical characteristics of breast cancer patients based on immunohistochemical biomarker.
**Table S2:** List of primers designed for RT‐PCR and microRNA Amplification.
**Table S3:** Other characteristics of the study population.
**Table S4:** Diagnostic performance of microRNA combinations based on ROC and AUC analyses obtained after LOOCV validation in breast cancer subtypes.
**Table S5:** Diagnostic performance of microRNA combinations based on ROC and AUC apparent analyses in breast cancer subtypes.
**Table S6:** Diagnostic performance of miR‐486‐3p and miR‐21‐5p combined with hormonal and proliferative markers obtained after LOOCV validation in differentiating luminal A and B subtypes.
**Table S7:** Apparent diagnostic performance of miR‐486‐3p and miR‐21‐5p combined with hormonal and proliferative markers in differentiating luminal A and B subtypes.

**Table S8:** Experimentally validated gene targets of hsa‐miR‐21‐5p identified using the multiMiR package.

**Table S9:** Experimentally validated gene targets of hsa‐miR‐486‐3p identified using the multiMiR package.

## Data Availability

The data supporting the findings of this study are available within the article and its Supplementary Material. Further details can be provided by the corresponding author upon reasonable request.
