# Supplementary material for: Exploratory Analysis of Circulating miR‐486‐3p and miR‐21‐5p in Early Detection and Subtype Classification of Luminal A and B Breast Cancer in Peruvian Women
Source: Cancer Rep (Hoboken). 2026 Oct 9;9(10):e70717. doi: 10.1002/cnr2.70717 (PMC13652960; doi:10.1002/cnr2.70717)
Supplement: Supplementary file 1 [file CNR2-9-e70717-s001.docx]

**Table S1.** Clinical Characteristics of Breast Cancer Patients Based on Immunohistochemical Biomarker

| **Biomarker** | **Category** | **n (%)** |
| --- | --- | --- |
| **PR** | Negative (< 20 %) | 18 (24) |
|  | Positive (≥ 20 %) | 57 (76) |
| **ER** | Negative (< 1%) | 0 (0.0) |
|  | Positive (≥ 1%) | 75 (100) |
| **HER2 Status** | Negative | 62 (82.7) |
|  | Positive | 13 (17.3) |
| **Ki-67 Proliferation Index** | Low proliferation (< 15%) | 22 (29.3) |
|  | High proliferation (≥ 15%) | 53 (70.7) |

PR: Progesterone receptor; ER: Estrogen receptor; HER2: Human epidermal growth factor receptor 2; Ki-67: proliferation index.

Cut-off values for ER/PR (≥ 20 %) and Ki-67 (≥ 15 %) were defined according to ASCO/CAP and St. Gallen guidelines, consistent with institutional diagnostic criteria at INEN.

**Table S2.** List of Primers Designed for RT-PCR and microRNA Amplification

| MicroRNA | Accession Number  (miRBase) | Forward 5’ →3’ | Reverse 5’ →3’ |
| --- | --- | --- | --- |
| hsa-miR-451a | MIMAT0001631 | CGCAGAAACCGTTACCA | GGTCCAGTTTTTTTTTTTTTTTAACTCAG |
| hsa-miR-660-5p | MIMAT0003338 | AGTACCCATTGCATATCGGA | GGTCCAGTTTTTTTTTTTTTTTCAAC |
| hsa-miR-21-5p | MIMAT0000076 | GCAGTAGCTTATCAGACTGATG | GGTCCAGTTTTTTTTTTTTTTTCAAC |
| hsa-miR-486-3p | MIMAT0004762 | GGGGCAGCTCAGTACA | GGTCCAGTTTTTTTTTTTTTTTATCCT |
| RT Primer | 5′-CAGGTCCAGTTTTTTTTTTTTTTTVN-3’ | | |

**Table S3.** Other Characteristics of the Study Population

|  | Control (n=36) n (%) | Luminal A (n=32)  n (%) | Luminal B (n=43)  n (%) | p-value | Test Used |
| --- | --- | --- | --- | --- | --- |
| Smoker | 1 (2.8%) | 0 (0.0%) | 2 (4.65%) | 0.776 | Fisher |
| Alcohol Consumption | 0 (0.0%) | 0 (0.0%) | 0 (0.0%) | 1 | Fisher |
| Menopause | 21 (58%) | 15 (47%) | 24 (56%) | 0.612 | Chi-square |
| Hormonal Treatment | 3 (8.3%) | 9 (28.1%) | 11 (25.58%) | 0.083 | Fisher |
| Other Treatment | 0 (0.0%) | 0 (0.0%) | 1 (2.33%) | 1 | Fisher |
| Family History of Breast Cancer | 2 (5.6%) | 21 (65.6%) | 24 (55.81%) | 1.042e-07 | Fisher |

n: number of individuals; p-value: statistical significance; Fisher: Fisher’s exact test. Statistical significance was set at p < 0.05.

**Table S4.** Diagnostic Performance of microRNA Combinations Based on ROC and AUC Analyses obtained after LOOCV validation in Breast Cancer Subtypes

| **Comparison** | **Positive Class** | **Combination** | **AUC (95% CI)** | **p-value** | **Threshold** | **Sensitivity** | **Specificity** | **PPV** | **NPV** | **Accuracy** |
| --- | --- | --- | --- | --- | --- | --- | --- | --- | --- | --- |
| **Control vs Luminal A** | **Luminal A** | miR21 | 0.625 (0.484–0.766) | 0.082 | 0.258 | 0.907 | 0.347 | 0.51 | 0.833 | 0.587 |
|  |  | miR486 | 0.651 (0.461–0.841) | 0.120 | 0.381 | 0.844 | 0.568 | 0.587 | 0.833 | 0.684 |
|  |  | miR21+miR486 | 0.590 (0.351–0.829) | 0.46 | 0.280 | 0.773 | 0.452 | 0.425 | 0.792 | 0.562 |
| **Control vs Luminal B** | **Luminal B** | miR21 | 0.301 (0.577–0.822) | 0.00146 | 0.476 | 0.446 | 0.986 | 0.971 | 0.634 | 0.712 |
|  |  | miR486 | 0.000 (0.000–0.000) | NA | 0.523 | 0.979 | 0.977 | 0.979 | 0.977 | 0.978 |
|  |  | miR21+miR486 | 0.635 (0.460–0.810) | 0.132 | 0.458 | 0.5 | 0.786 | 0.7 | 0.611 | 0.643 |
| **Luminal A vs Luminal B** | **Luminal B** | miR21 | 0.538 (0.388–0.689) | 0.617 | 0.578 | 0.635 | 0.648 | 0.712 | 0.565 | 0.641 |
|  |  | miR486 | 0.681 (0.500–0.863) | 0.0505 | 0.644 | 0.562 | 0.844 | 0.844 | 0.562 | 0.675 |
|  |  | miR21+miR486 | 0.690 (0.466–0.914) | 0.0959 | 0.647 | 0.738 | 0.682 | 0.816 | 0.577 | 0.719 |

AUC: Area Under the Curve, PPV: Positive Predictive Value, NPV: Negative Predictive Value, miR: microRNA, All reported AUCs were statistically significant (DeLong’s test, p < 0.05), LOOCV = leave-one-out cross-validation performance with 95% CI.

**Table S5.** Diagnostic Performance of microRNA Combinations Based on ROC and AUC Apparent Analyses in Breast Cancer Subtypes

| **Comparison** | **Positive Class** | **Combination** | **AUC (95% CI)** | **p-value** | **Threshold** | **Sensitivity** | **Specificity** | **PPV** | **NPV** | **Accuracy** |
| --- | --- | --- | --- | --- | --- | --- | --- | --- | --- | --- |
| **Control vs Luminal A** | **Luminal A** | miR21 | 0.677 (0.542–0.812) | 0.0104 | 1.002 | 0.981 | 0.347 | 0.53 | 0.962 | 0.619 |
|  |  | miR486 | 0.717 (0.540–0.895) | 0.0166 | -0.025 | 0.844 | 0.614 | 0.614 | 0.844 | 0.711 |
|  |  | miR21+miR486 | 0.720 (0.499–0.941) | 0.0509 | 0.319 | 0.773 | 0.643 | 0.531 | 0.844 | 0.688 |
| **Control vs Luminal B** | **Luminal B** | miR21 | 0.454 (0.315–0.593) | 0.516 | 0.220 | 0.527 | 0.625 | 0.591 | 0.562 | 0.575 |
|  |  | miR486 | 0.499 (0.320–0.678) | 0.991 | -0.370 | 0.688 | 0.477 | 0.589 | 0.583 | 0.587 |
|  |  | miR21+miR486 | 0.600 (0.419–0.781) | 0.279 | 0.459 | 0.833 | 0.452 | 0.603 | 0.731 | 0.643 |
| **Luminal A vs Luminal B** | **Luminal B** | miR21 | 0.627 (0.486–0.768) | 0.0774 | -0.723 | 0.662 | 0.648 | 0.721 | 0.583 | 0.656 |
|  |  | miR486 | 0.762 (0.596–0.928) | 0.00195 | 0.261 | 0.771 | 0.719 | 0.804 | 0.676 | 0.75 |
|  |  | miR21+miR486 | 0.790 (0.574–1.000) | 0.00767 | 0.659 | 0.833 | 0.773 | 0.875 | 0.708 | 0.812 |

AUC: Area Under the Curve. PPV: Positive Predictive Value. NPV: Negative Predictive Value, miR: microRNA, Apparent = raw model performance without cross-validation.

**Table S6.** Diagnostic Performance of miR-486-3p and miR-21-5p Combined With Hormonal and Proliferative Markers obtained after LOOCV validation in Differentiating Luminal A and B Subtypes

| **Comparison** | **Combination** | **AUC**  **(95% CI)** | **p-value** | **Threshold** | **Sensitivity** | **Specificity** | **PPV** | **NPV** | **Accuracy** |
| --- | --- | --- | --- | --- | --- | --- | --- | --- | --- |
| **Luminal A vs Luminal B** | miR486 + miR21 | 0.690 (0.466–0.914) | 0.0959 | 0.647 | 0.738 | 0.682 | 0.816 | 0.577 | 0.719 |
|  | Ki-67 | 0.855 (0.649–1.000) | 0.0000736 | 0.524309 | 0.929 | 0.864 | 0.929 | 0.864 | 0.906 |
|  | miR486 + miR21 + Ki-67 | 0.785 (0.551–1.000) | 0.0128 | 0.347123 | 0.929 | 0.773 | 0.886 | 0.85 | 0.875 |
|  | miR486 + miR21 + HER2 + PR + ER | 0.660 (0.412–0.908) | 0.206 | 0.673116 | 0.69 | 0.773 | 0.853 | 0.567 | 0.719 |
|  | miR486 + miR21 + Ki-67 + HER2 + PR + ER | 0.670 (0.414–0.926) | 0.193 | 0.134149 | 0.881 | 0.682 | 0.841 | 0.75 | 0.812 |
|  | Ki-67 + HER2 + PR + ER | 0.750 (0.510–0.990) | 0.0411 | 0.439891 | 0.881 | 0.682 | 0.841 | 0.75 | 0.812 |

AUC: Area Under the Curve. PPV: Positive Predictive Value. NPV: Negative Predictive Value. ER: Estrogen Receptor, PR: Progesterone Receptor, HER2: Human Epidermal Growth Factor Receptor 2, Ki-67: Cellular proliferation marker. LOOCV = leave-one-out cross-validation performance with 95% CI. Statistical significance was set at p < 0.05.

**Table S7.** Apparent Diagnostic Performance of miR-486-3p and miR-21-5p Combined With Hormonal and Proliferative Markers in Differentiating Luminal A and B Subtypes

| **Comparison** | **Combination** | **AIC** | **AUC**  **(95% CI)** | **p-value** | **Threshold** | **Sensitivity** | **Specificity** | **PPV** | **NPV** | **Accuracy** |
| --- | --- | --- | --- | --- | --- | --- | --- | --- | --- | --- |
| **Luminal A vs Luminal B** | miR486 + miR21 | 38.5 | 0.790 (0.574–1.000) | 0.00767 | 0.659 | 0.833 | 0.773 | 0.875 | 0.708 | 0.812 |
|  | Ki-67 | 18.11 | 0.932 (0.835–1.000) | 0 | 0.520592 | 0.929 | 0.864 | 0.929 | 0.864 | 0.906 |
|  | miR486 + miR21 + Ki-67 | 18.41 | 0.980 (0.942–1.000) | 0 | 0.673623 | 0.929 | 0.864 | 0.929 | 0.864 | 0.906 |
|  | miR486 + miR21 + HER2 + PR + ER | 37.45 | 0.855 (0.714–0.996) | 7.48E-07 | 0.553137 | 0.833 | 0.773 | 0.875 | 0.708 | 0.812 |
|  | miR486 + miR21 + Ki-67 + HER2 + PR + ER | 21.7 | 0.990 (0.966–1.000) | 0 | 0.672577 | 0.929 | 0.955 | 0.975 | 0.875 | 0.938 |
|  | Ki-67 + HER2 + PR + ER | 19.32 | 0.985 (0.955–1.000) | 0 | 0.803693 | 0.881 | 0.955 | 0.974 | 0.808 | 0.906 |

AUC: Area Under the Curve. PPV: Positive Predictive Value, NPV: Negative Predictive Value, ER: Estrogen Receptor, PR: Progesterone Receptor, HER2: Human Epidermal Growth Factor Receptor 2, Ki-67: Cellular proliferation marker, AIC: Akaike Information Criterion (model quality metric), Apparent = raw model performance without cross-validation.Statistical significance was set at p < 0.05.
