# Supplementary material for: Exploratory Analysis of Circulating miR‐486‐3p and miR‐21‐5p in Early Detection and Subtype Classification of Luminal A and B Breast Cancer in Peruvian Women
Source: Cancer Rep (Hoboken). 2026 Oct 9;9(10):e70717. doi: 10.1002/cnr2.70717 (PMC13652960; doi:10.1002/cnr2.70717)
Supplement: Supplementary file 2 [file CNR2-9-e70717-s003.docx]

**Figure S1.** Representative melt-curve analysis of the RT-qPCR assays. Melt curves were generated for (A) miR-21-5p, (B) miR-451a, (C) miR-486-3p, (D) miR-660-5p, and (E) RNU6. Each curve represents individual samples included in a representative qPCR plate. A predominant melting peak was observed for each target. Melt-curve analysis was performed for all qPCR runs.

G

**Figure S2.** Comparison of Non-Genetic Factors Between Breast Cancer Subtypes and the Control Group. (A) Boxplot comparing BMI across the Control, Luminal A, and Luminal B groups. (B) Boxplot showing the distribution of age among groups. (C) Boxplot comparing the age at menarche. (D) Boxplot illustrating differences in age at first pregnancy. (E) Boxplot comparing the number of pregnancies between groups. (F) Bar plot showing the proportion of individuals with a family history of cancer across groups. (G) Bar chart showing the proportion of postmenopausal women among different groups.

**Figure S3.** Spearman correlation heatmaps between circulating miRNA expression levels and clinico-pathological/lifestyle variables across breast cancer subtypes and controls. (A) Luminal A group; (B) Luminal B group; (C) Control group; (D) Combined Luminal A + B groups. The color scale represents the strength and direction of the correlation (red = positive, blue = negative). Correlations were computed using Spearman’s rank method. Binary variables such as Smoker and Family History of Cancer are shown for completeness, but their interpretation is limited by small sample sizes (e.g., only three smokers).

**Figure S4.** Evaluation of AUC values for predictive models based on miR-21-5p and miR-486-3p (apparent performance, before validation). (A) Control vs. Luminal A, (B) Control vs. Luminal B, and (C) Luminal A vs. Luminal B.

**Figure S5**. ROC curve analysis comparing the predictive performance of miR-486-3p and miR-21-5p combined with HER2, PR, ER, and Ki-67 in distinguishing Luminal A from Luminal B breast cancer subtypes. (p < 0.05)
